# Recurrent primary intracranial synovial sarcoma, a case report and review of the literature

**DOI:** 10.1002/ccr3.6273

**Published:** 2022-09-05

**Authors:** Ataee Kachuee Manizhe, Iman Mohseni, Alireza Sahranavard, Zhale Tabrizi

**Affiliations:** ^1^ Department of Radiology, Firouzgar Hospital Iran university of medical science Tehran Iran; ^2^ Department of orthopaedics surgery, Alzahra hospital Isfahan university of medical science Isfahan Iran; ^3^ Department of Radiology Iran university of medical science Tehran Iran

**Keywords:** metastatic intracranial synovial sarcoma, primary intracranial synovial sarcoma, synovial sarcoma

## Abstract

Synovial sarcoma (SS) occurs in various parts of the body, predominantly in the extremities. It also occurs in organs without synovial structures. The intracranial disease has been reported as metastasis, but primary intracranial SS has been reported rarely. We report a patient with hemiplegia and a mass on the brain CT. Pathology showed SS with no extracranial pathology.

## Abstract 2

Synovial sarcoma (SS) is one of the most common sarcomas of soft tissue, and it mostly occurs in the lower extremities. It can cause metastasic disease, especially in the lungs, bones, and lymph nodes. Metastasis in the brain is very rare. Primary intracranial SS has also been reported in extremely rare cases. In this article, we study the case of a 28‐year‐old man with primary intracranial SS.

## 1 INTRODUCTION

Synovial sarcoma (SS) is the fourth most common sarcoma of the soft tissue, following liposarcoma, pleomorphic undifferentiated sarcoma, and rhabdomyosarcoma. With more prevalence in men than women. Most of the SS cases are located in extremities, predominantly lower extremities^1^, ^2^, ^3^, especially near large joints around the knee and thigh.^4^ SS has an annual incidence of 2.5 per 100,000.^5^ Intracranial SS is very uncommon and has been reported as a metastasis lesion from SS in other parts of the body.^4^, ^6^, ^7^, ^8^, ^9^, ^10^, ^11^, ^12^ In this study, we report a case with no obvious primary extracranial pathology, with recurrent primary intracranial SS, which has been very rarely reported in the literature.

## 2 CASE PRESENTATION

**FIGURE 1:**
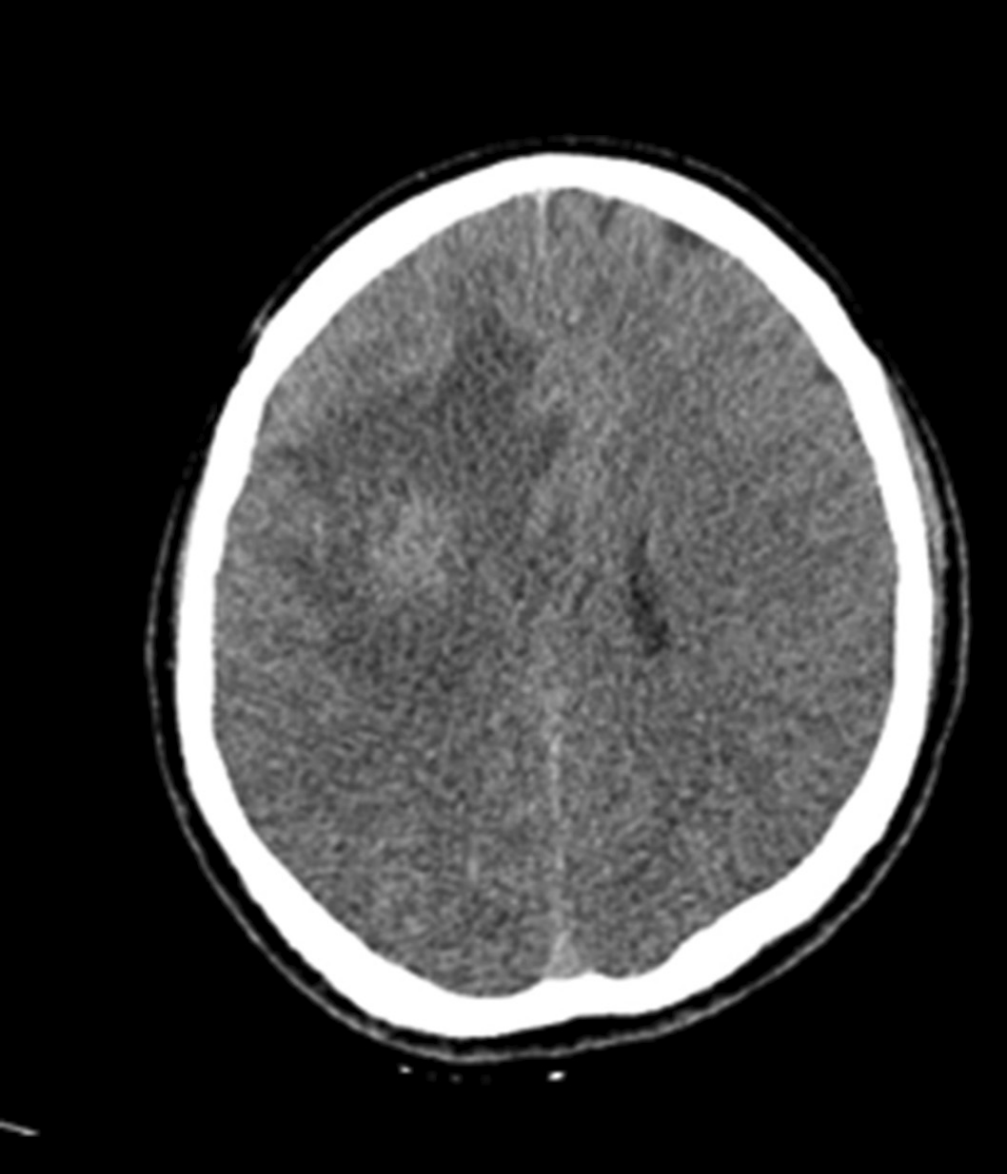
Brain computed tomography (CT) scan revealed an intra axial mass lesion with the central necrotic component and surrounding vasogenic edema in the right frontal white matter causing midline shift to the left side

**FIGURE 2:**
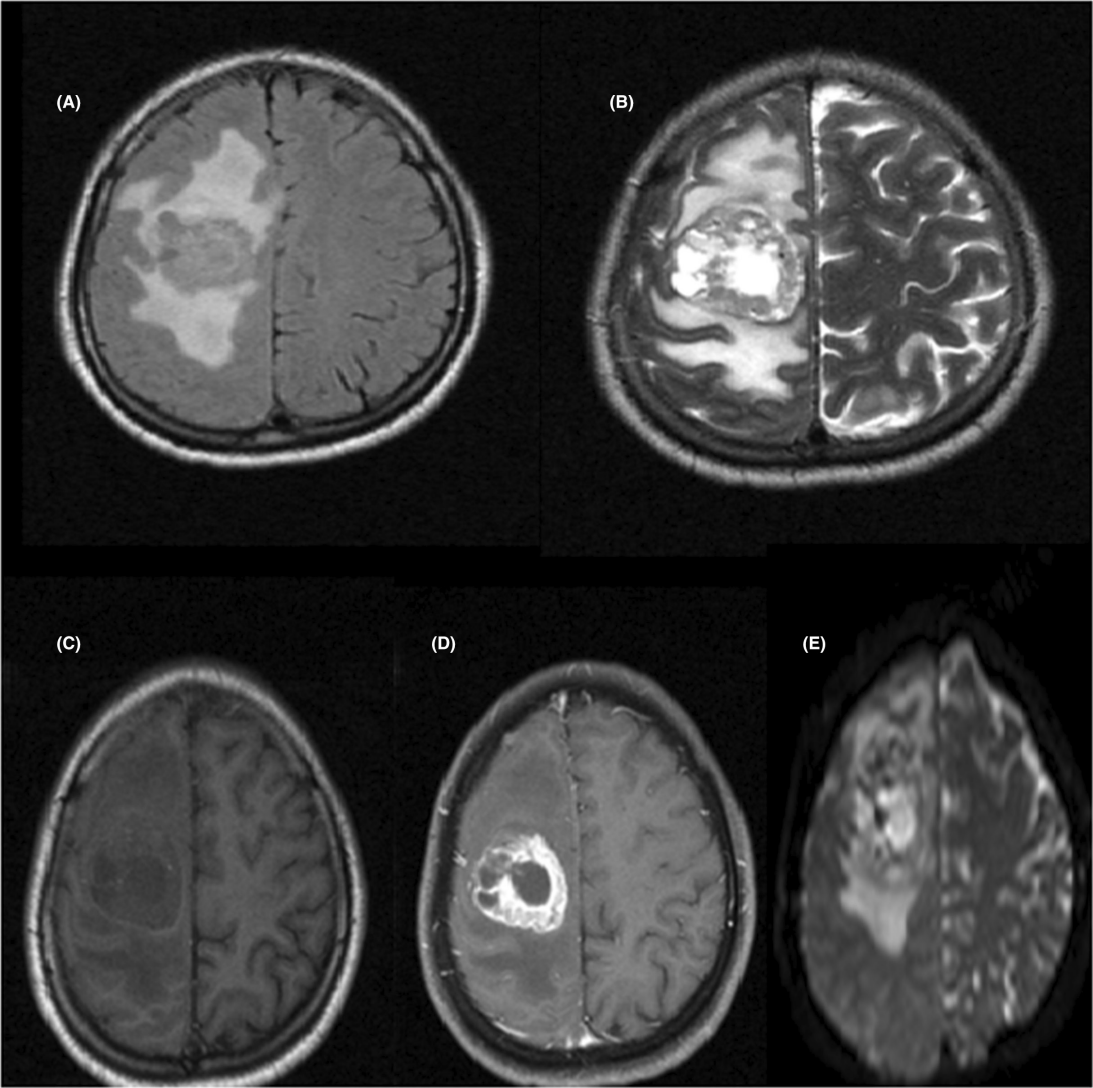
Magnetic resonance imaging (MRI) without gadolinium contrast. A:T2 Flair Axial sequence. B:T2 Axial sequence. The above images show an intra axial well‐circumscribed heterogenous mass with central necrosis and surrounding vasogenic edema in right frontal white matter. Magnetic resonance imaging (MRI) with gadolinium contrast C:T1 Axial sequence without gadolinium contrast.D:T1 with gadolinium contrast.E:DWI sequence. The above images show an Intra axial well‐circumscribed heterogenous mass with central necrosis and irregular peripheral enhancement and significant surrounding vasogenic edema in right frontal white matter. restriction of the peripheral solid component of mass was also seen

The case study is devoted to investigating headache and left hemiplegia in a 28‐year‐old man 10 days before coming to the emergency room. Neurological examination revealed left‐sided hemiplegia, without gait instability and ataxia. Brain computed tomography (CT) scan revealed an intra axial mass lesion with a central necrotic component and surrounding vasogenic edema in the right frontal white matter causing a midline shift to the left side (Figure 1). The patient underwent magnetic resonance imaging (MRI) with gadolinium contrast for more evaluation, and the findings were as mentioned below: an intra axial well‐circumscribed heterogeneous mass with central necrosis and irregular peripheral enhancement and significant surrounding vasogenic edema in the right frontal white matter. Restriction of the peripheral solid component of mass was also seen (Figure 2).

**FIGURE 3:**
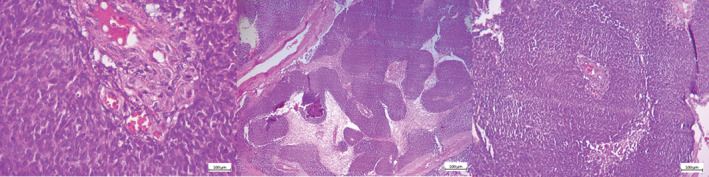
The histopathological assessment of the mass shows neoplastic tissue composed of biphasic pattern, large irregular sheets of monotonous cells, some with central necrosis set in fibroblastic stroma. Tumoral cells are plump and have very high N/C ratio. Frequent mitotic figures are present

The patient was taken to the operation room and underwent total excision of the mass. Many hours later the left hemiplegia gradually improved. The histopathological assessment of the mass revealed neoplastic tissue composed of biphasic pattern: large irregular sheets of monotonous cells some with central necrosis set in the fibroblastic stroma. Tumoral cells are plump and have a very high N/C ratio. Frequent mitotic figures are present (Figure 3).

**FIGURE 4:**
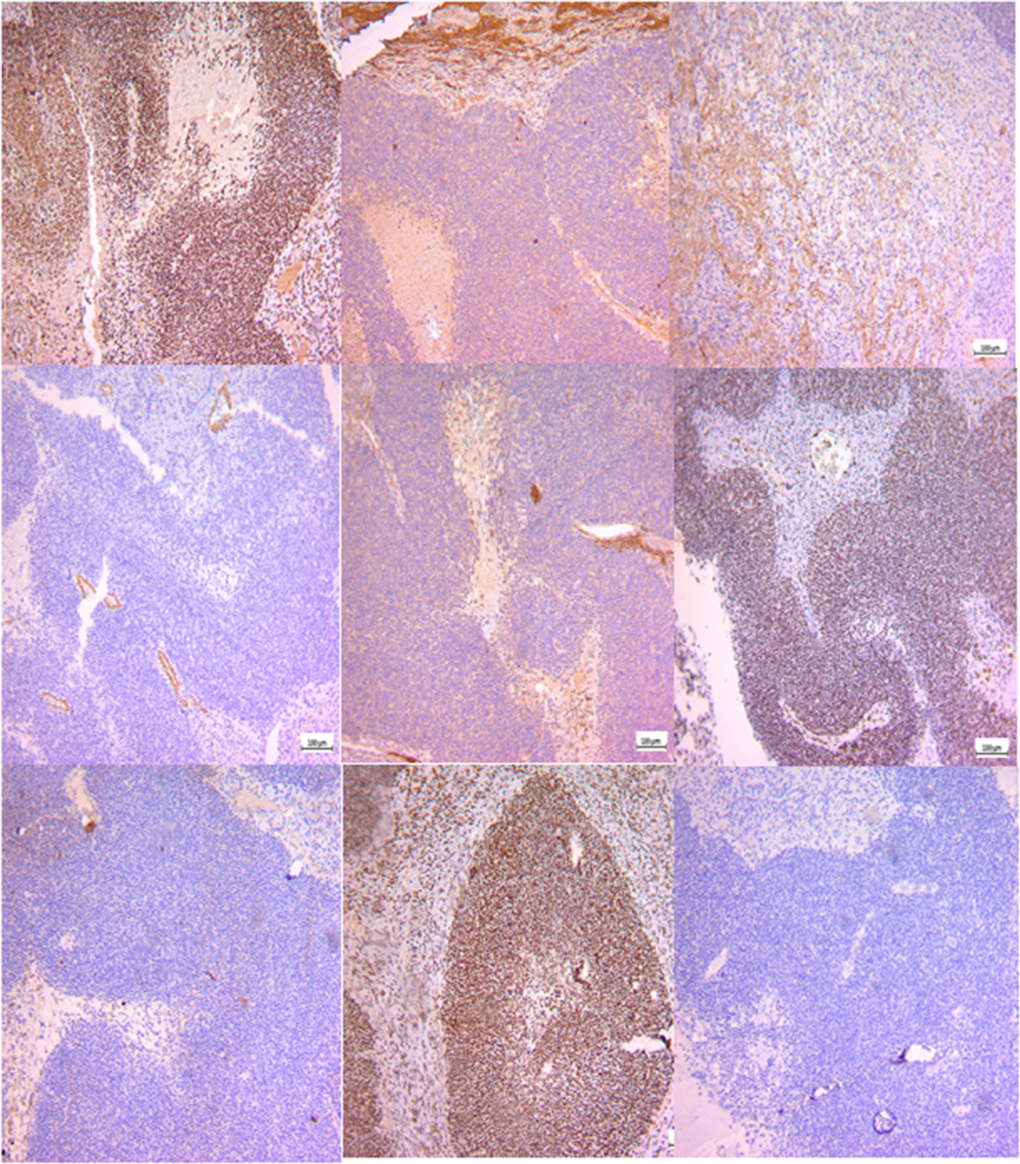
IHC staining shows positive reactivity for TLE1, FLI1, and INI1 and negative reactivity for Synaptophysin, Olig 2, SMA, CK and CD 99. The above findings are consistent with SS.

IHC staining shows positive reactivity for TLE1, FLI1, and INI1 and negative reactivity for Synaptophysin, Olig 2, SMA, CK and CD 99. GFAP staining is nonspecific (Figure 4). The above findings are consistent with SS. The abdominopelvic CT scan was normal. Also,the PET scan did not show any other metastasis.

The patient was discharged 1 week after surgery without any neurological problems.

He underwent radiotherapy 1 month after his discharge.

**FIGURE 5:**
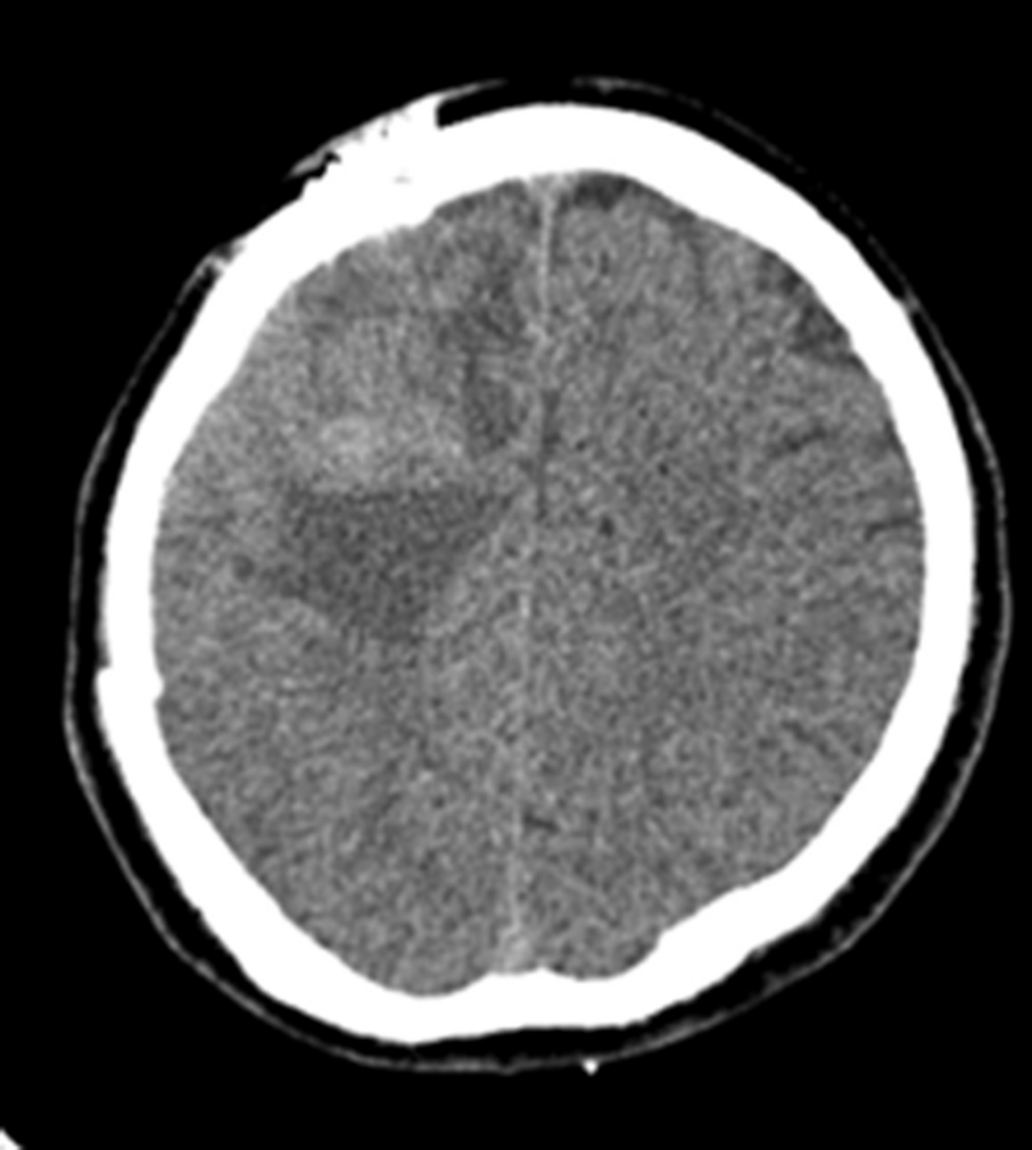
Brain CT scan showed recurrence of the previous tumor

Four months later, the patient came back to the neurology emergency room with left hemiplegia, he underwent CT scan findings that revealed a recurrence of the previous tumor (Figure 5), so he was taken to the operating room again and underwent total excision of the mass. The histopathological assessment showed SS recurrence. PET scan was done again and there was no evidence of primary SS anywhere else or any other metastasis. The patient was discharged 1 week later without any neurological signs or symptoms.

## 3 DISCUSSION

SS is a form of soft tissue sarcoma that occurs mostly in young and middle‐aged adults and has more tendency to occur in extremities, however, it can occurs in children and old individuals and involve other parts of the body.^13^, ^14^ It is believed that 5–10 percent of soft tissue sarcomas are SS. The tumor is closely related to a tendon sheath, tendon, joint capsule, and bursal structures.^15^ It may also arise primarily in other parts of the body, such as the heart, kidney, and lungs.^4^ Synovial cell sarcomas arise from type B synoviocytes.^16^ There is an alternative sarcoma subtype, which originates from type A synoviocytes, called histiocytic sarcoma.^17^ There are four subtypes of SS, as mentioned below: monophasic, monophasic epithelial, biphasic, and poorly differentiated (round cell tumors).^4^, ^18^ Classical SS has a biphasic morphology and its pathological findings are sheets of spindle cells and sharply segregated epithelial cells forming gland‐like areas. The second form of SS is monophasic containing only a sarcomatous component. Most of the SSs are of these two forms mentioned earlier (4)IHC staining can be positive for epithelial markers (keratin and EMA) and CD99, S‐100 protein, and BCL2. TLE1 is the most sensitive and specific marker for SS.^19^ Because the tumor resembled synovial tissue under the light microscope, it was named “Synovial Sarcoma” by Sabrazes in 1934.^20^ However, the cellular origin of this tumor is possibly from neural crest‐derived malignant peripheral nerve cells.^4^ Chromosomal translocation of t(X;18)(p11.2;q11.2) can be investigated in over 95% of SS.^5^ the treatment of choice for primary tumors is excisional surgery, while metastatic SS cannot be treated with excisional surgery but chemotherapy and radiotherapy may help them.^21^ In addition, the current understanding of cancer cells has highlighted the importance of mitochondria in cancer biogenesis.^22^ This issue has been found specifically in SSs.^23^ Besides, considering the 75% expression of PD‐L1 on SS cells, one might assume that this modality might be applied in the future for primary intracranial SSs.^24^ Current literature has noted that the immunotherapy results can be improved by improving mitochondria biogenesis.^25^In some patients, SS is diagnosed when it reaches out to other organs such as lungs, bone, and lymph nodes.^6^, ^9^, ^26^ Intracranial SS is very rare and in most cases is reported as a metastasis from SS.^27^ SS presenting as an intracranial has been reported very rarely, as mentioned below:

1. Kleinschmidt‐DeMasters et al reported the case of a 19‐year‐old woman with primary synovial sarcoma of the third ventricle,^28^
2. Bettio et al reported a 36‐year‐old man with a large intracranial tumor involving the cranial base, with the invasion of the sellar region and sphenoidal sinus.^29^
3. Mohit Patel et al reported a primary intracranial synovial sarcoma as a right parietal heterogeneous, hyperdense mass with a large medial hematoma in a 21‐year‐ old man.^27^
4. Yang‐Yang Wang et al reported a 35‐year‐old man with left intracerebral lesions with hemorrhage and left middle cerebral artery arteriovenous malformation, respectively.^30^

The clinical manifestations of primary intracranial SS are nonspecific, such as headache, nausea, vomiting, and hemiplegia. Radiologic features of primary SS are also nonspecific and the final diagnosis is made by pathological and immunohistochemistry features.^30^

Our patient was a 28‐year‐old man with a headache and left hemiplegia. Neurological examination revealed left‐sided hemiplegia, without gait instability and ataxia. Brain computed tomography (CT) scan revealed an intra axial mass lesion with a central necrotic component and surrounding vasogenic edema in right frontal white matter causing midline shift to the left side. The patient underwent MRI with gadolinium contrast for more evaluation, and the findings were as mentioned below: An Intra axial well‐circumscribed heterogeneous mass with central necrosis and irregular peripheral enhancement and significant surrounding vasogenic edema in right frontal white matter. Restriction of the peripheral solid component of mas was also seen. The patient was taken to the operation room and underwent total excision of the mass. Many hours later, the left hemiplegia was gradually improved. The histopathological assessment of the mass revealed neoplastic tissue composed of biphasic patterns: large irregular sheets of monotonous cells some with central necrosis set in the fibroblastic stroma. Tumoral cells are plump and have a very high N/C ratio. Frequent mitotic figures are present.

IHC staining show positive reactivity for TLE1, FEI1, and INI1 and negative reactivity for Synaptophysin,Olig 2, SMA, CK CD 99. GFAP staining is nonspecific. The aforementioned findings are consistent with SS. The PET scan did not show any other metastatic disease. The abdominopelvic CT scan was also normal.

## 4 CONCLUSION

SS is one of the most common sarcomas of soft tissue, and it mostly occurs in the lower extremities. It can cause metastasic disease, especially in the lungs, bones, and lymph nodes. Metastasis in the brain is very rare. Primary intracranial SS has also been reported extremely rare. In this article, we present a 28‐year‐old man with primary intracranial SS. It can be misdiagnosed with other intracranial masses. So, it is important to confirm the diagnosis with pathology and resect the tumor and start radiotherapy treatment if needed.

## AUTHOR CONTRIBUTION

MAK and IM chose the case, reported the CT scan and MRI and supervised manuscript preparation. ZHT reviewed the published literature and wrote the case report manuscript. AS reviewed the published literature, interviewed the patient, and collected the patient past medical history. MAK, IM, and AS edited the written manuscript. All the authors read and approved the final manuscript.

## CONFLICT OF INTEREST

The authors declare that there are no competing interests.

## ETHICAL APPROVAL

All the ethics standards and consent were obtained for this manuscript.

## CONSENT

Written informed consent for publication was obtained from the patient.

## Data Availability

Data sharing not applicable ‐ no new data generated Data sharing is not applicable to this article as no new data were created or analyzed in this study.
